# Developing safe and efficient CGBE editor based on Cas-embedding strategy

**DOI:** 10.1016/j.synbio.2025.02.001

**Published:** 2025-02-06

**Authors:** Tian Lin, Xin Wang, Yu Zhang, Guanglei Li, Xingxu Huang, Ming Shi

**Affiliations:** a Cancer Institute, Xuzhou Medical University, 209 Tongshan Road, Xuzhou, Jiangsu, 221004, China; b Center of Clinical Oncology, The Affiliated Hospital of Xuzhou Medical University, 99 Huaihai Road, Xuzhou, Jiangsu, 221002, China; c Jiangsu Center for the Collaboration and Innovation of Cancer Biotherapy, Xuzhou Medical University, 209 Tongshan Road, Xuzhou, Jiangsu, 221004, China; d Gene Editing Center, School of Life Science and Technology, ShanghaiTech University, Shanghai, 201210, China; e Shanghai Institute for Biomedical and Pharmaceutical Technologies, Shanghai, 200032 China

**Keywords:** CGBE, Cas-embedding, Off-target, eA3A

## Abstract

CGBE (C-to-G base editor) systems, pivotal components within the base editing arsenal, enable the precise conversion of cytosines to guanines. However, conventional cytidine deaminases possess non-specific single-stranded DNA binding properties, leading to off-target effects and safety concerns. The Cas-embedding strategy, which involves embedding functional proteins like deaminases within the Cas9 enzyme's architecture, emerges as a method to mitigate these off-target effects. Our study pioneers the application of the Cas-embedding strategy to CGBE systems, engineering a suite of novel CGBE editors, CE-CGBE. The CE-CGBE that incorporated eA3A, RBMX and Udgx excelled in editing efficiency, editing purity, and indel formation was named HF-CGBE. HF-CGBE showed no significant difference in off-target effects compared to the negative control group for both DNA and RNA. In summary, the novel HF-CGBE editors we propose expand the base editing toolbox and provide therapeutic approaches for related pathogenic mutations.

## Graphical abstract

**Figure:**
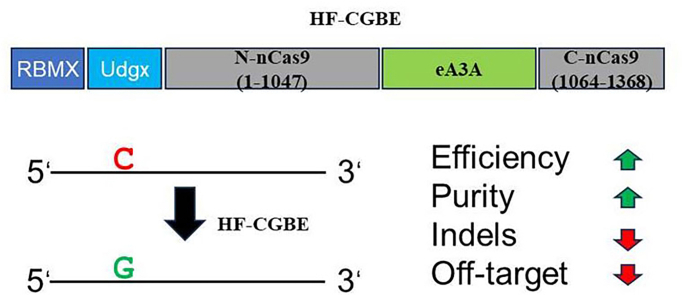

## 1 Introduction

The first gene editing product, Casgevy, was approved for market entry in November 2023 [1]. It utilizes CRISPR/Cas9 to cut the BCL11A gene, thereby inhibiting its activity [2]. However, the first gene editing product is not without flaws, as it causes double-strand breaks (DSBs) in DNA, leading to unpredictable genotypes upon repair. Therefore, CRISPR2.0, which does not rely on DSBs, will be a focus for future development [3]. Currently, base editing, prime editing, and epigenome editing are three major research hotspots. Base editing, in particular, stands out due to its efficiency and ease of use, with several products leveraging base editing for clinical trials [4]. Base editing is expected to become the next generation of approved products.

Current base editing primarily includes cytosine base editing and adenine base editing [5]. Additionally, various other types of base editors are continuously being optimized, such as CGBE [6,7]. CGBE editors are derived from cytosine base editing technology and can achieve C-to-G transversions. Tan et al. constructed rAPOBEC-nCas9-rXRCC1 CGBE by fusing the base excision repair protein XRCC1 [8]. Koblan et al. screened multiple factors affecting CGBE editing efficiency using a library approach [9]. Kurt et al. developed CGBE1 and miniCGBE1 by fusing UNG from *E. coli* [10]. Yuan et al. developed OPTI-CGBEs through optimizing the position of functional proteins and codon optimization [11]. Zhao et al. developed GBE editors using AID deaminase, and subsequently, the same team improved editing efficiency and purity by fusing Rad51 to develop GBE2.0 [7].

Current optimizations of CGBE primarily aim to improve editing efficiency and purity, but off-target effects of CGBE also require attention. The CGBE is a CBE from which UGI has been removed, utilizing cytidine deaminases for deamination editing [10]. However, CBE exhibit sgRNA-independent off-target effects at both the DNA and RNA levels [12,13]. Therefore, reducing off-target effects of CGBE is an important aspect alongside pursuing efficiency and purity. For CBE, various methods have been developed to reduce off-targeting, such as mutating the deaminase, splitting the editing system into two parts that must act together to achieve deamination editing at the target site [13,14]. Previously, we identified flexible regions within Cas9 using a library screening approach, indicating that the deaminase can be integrated as an independent functional domain with Cas9 [15]. This integration serves two purposes: first, it restricts the deaminase's activity, preventing it from randomly binding to ssDNA within the cell and causing off-target effects. Secondly, this fusion can make the resulting protein more stable, facilitating large-scale purification and stable storage of the associated proteins, thus making it more applicable for use in gene and cell therapy products [4].

Building on previous work, this study developed a new class of base editors, CE-CGBE (Cas-embedding CGBE), by fusing different types of cytidine deaminases with Cas9 using a Cas-embedding strategy. We further integrated functional proteins that influence C-to-G editing efficiency and ultimately optimized a highly efficient, pure, and low-off-target HF-CGBE. The optimized HF-CGBE will further expand the base editing toolkit and has the potential to bring significant value in areas such as cell therapy, genetic disease correction, and prevention of infectious diseases.

## 2 Results

### 2.1 CGBE base editors and their cas-embedding variants based on different types of deaminases

**Fig. 1:**
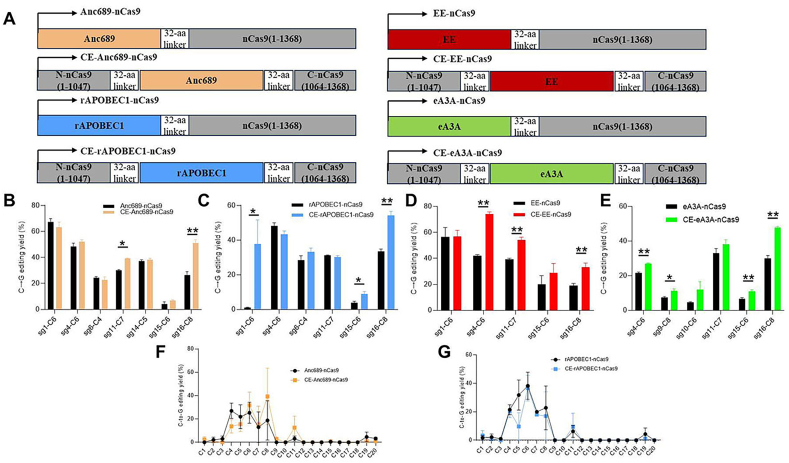
**Different Types of Deaminase-Mediated CGBE Editors.** (A) Four different types of deaminases, including Anc689, rAPOBEC1, EE, and eA3A, were combined with Cas9 to form CGBE editors. One approach involved direct fusion constructs, while another involved fusing the deaminase to the middle position of Cas9, replacing residues 1048–1063. (B) Comparison of editing efficiency for C-to-G conversions between Anc689-nCas9 and CE-Anc689-nCas9. The highest efficiency point was recorded at each site. ∗ indicates significant differences, ∗∗ indicates highly significant differences. Each sample was replicated three times. The statistical method used was Student's two-sided *t*-test. (C–E) Editing efficiencies for editors mediated by rAPOBEC1, EE, and eA3A, respectively. (F) Statistical analysis of the C-to-G editing window for editors mediated by Anc689. (G) Statistical analysis of the C-to-G editing window for editors mediated by rAPOBEC1. The protospacer is at positions 1–20, with the SpCas9 PAM at positions 21–23.

CGBE base editors are optimized from cytidine base editors, which fuse a cytidine deaminase, nCas9, and UGI. If the UGI is removed from cytidine base editors, intracellular UNG will cleave the deaminated cytidine, forming an apyrimidinic (AP) site [16]. With DNA repair, this leads to C mutating to G. At this point, the structure of the CGBE base editor is similar to that of the CBE base editor, and its cytidine deaminase can still randomly contact ssDNA within the cell, causing off-target effects. Studies have shown that Cas9 contains flexible regions, and placing the deaminase in these flexible regions can reduce off-target effects [15,17]. Previously, we identified flexible regions within Cas9 using a random library approach and developed a safe and efficient base editing system using a Cas-embedding strategy [15]. To investigate the value of this strategy in CGBE editors, we constructed CGBE and CE-CGBE editors mediated by different deaminases. The four deaminases used are Anc689 [18], rAPOBEC1 [19], EE [19], and eA3A [20], and their corresponding CGBE editors are named: Anc689-nCas9, rAPOBEC1-nCas9, EE-nCas9, and eA3A-nCas9, respectively. Additionally, we replaced residues 1048aa-1063aa of nCas9 with the deaminase to construct corresponding Cas-embedding CGBEs, named CE-Anc689-nCas9, CE-rAPOBEC1-nCas9, CE-EE-nCas9, and CE-eA3A-nCas9 (Fig. 1A).

Eight types of C-to-G base editors along with their respective sgRNAs were transfected into HEK293T cells. The sgRNA expression vector contains the GFP gene, which serves as a signal for sorting. GFP positive cells were sorted using flow cytometry, and then the DNA was extracted. Deep sequencing was used to analyze the C-to-G editing efficiency, editing purity, and the proportion of indels. The results showed that for Anc689-nCas9 and its corresponding CE-Anc689-nCas9, out of seven editing sites, there was no significant change in editing efficiency at five sites, while at one site, the editing efficiency increased from 26.5 % to 60 % (Fig. 1B). We also observed that the editing efficiency of base editor varied significantly across different sites, ranging from as low as around 4 % to nearly 70 %. Similar variations were seen in the editing efficiencies of rAPOBEC1, EE, and eA3A CGBE editors and their corresponding Cas-embedding editors (Fig. 1C–E). Among the four CE-CGBEs, CE-eA3A-nCas9 showed the greatest improvement in efficiency. At four out of the six tested loci, the editing efficiency of CE-eA3A-nCas9 was significantly higher compared to the control (Fig. 1E). Further analysis of the editing windows of C-to-G base editors showed that the editing window of CE-CGBE did not show significant improvement compared to controls (Fig. 1F and G).

**Fig. 2:**
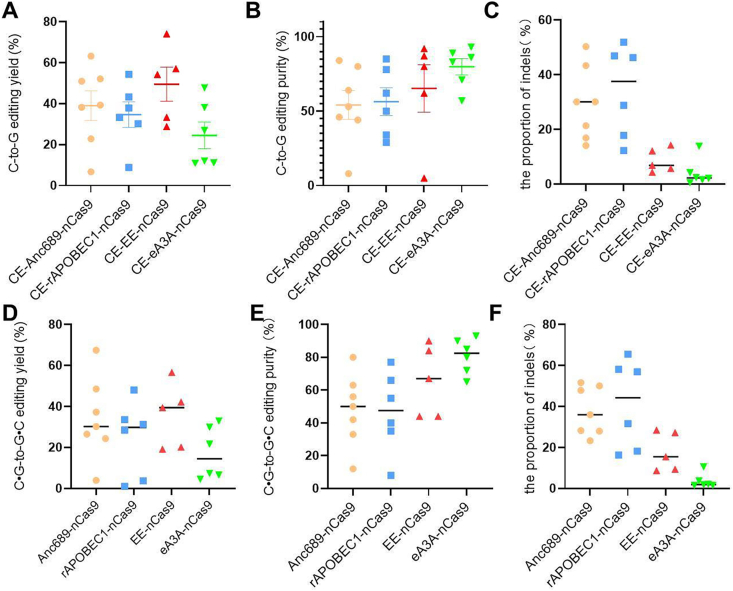
**Analysis of Editing Efficiency, Purity, and Indel Statistics for Different Cas-Embedding CGBE Base Editing Systems.** (A) Analysis of editing efficiency for different Cas-embedding CGBE base editing systems, and the values in the graphs are mean ± s.e.m. (B) Analysis of editing purity for different Cas-embedding CGBE base editing systems, and the values in the graphs are mean ± s.e.m. (C) Analysis of indels for different Cas-embedding CGBE base editing systems, and the values in the graphs are mean ± s.e.m. (D–F) Analysis of editing efficiency (D), purity (E) and indels (F) for traditional CGBEs, and the values in the graphs are mean ± s.e.m.

Next, we analyzed the purity of C-to-G editing for eight types of base editors, which refers to the proportion of C-to-G editing out of total editing events. We found that for CGBEs mediated by Anc689 (Fig. S1A), rAPOBEC1 (Fig. S1B), and EE (Fig. S1C) deaminases, as well as their Cas-embedding counterparts, the editing purity varied significantly across different sites, with trends closely matching those of editing efficiency. For C-to-G editing purity mediated by eA3A, the purity reached relatively high levels, with four out of six sites achieving over 80 % purity, and the lowest purity being 55 % (Fig. S2D). For the four CE-CGBE editors, we analyzed the editing efficiency and editing purity across different target sites uniformly. We found that the CE-CGBE based on EE had the highest average editing efficiency (Fig. 2A). However, for editing purity, the editor based on eA3A achieved an average purity of 80 % (Fig. 2B). The four conventional types of CGBE share similar characteristics regarding editing efficiency and editing purity (Fig. 2D and E).

Subsequently, we analyzed the efficiency of indel generation. The results indicated that three of CE-CGBE editors reduced indels at most sites compared to the CGBE editors (Figs. S1E–S1G). However, for the CE-CGBE editors dependent on eA3A, there was no significant change in indel generation compared to CGBE (Fig. S1H). Yet, the CE-CGBE based on eA3A had the lowest indel generation among the four CE-CGBEs (Fig. 2C). A similar trend is also evident in the four conventional CGBE editors (Fig. 2F). Taken together, although the C-to-G editing efficiency of the eA3A-based editor is not the highest, its editing purity and indel generation are the best. Precision is a critical factor in evaluating editors; therefore, we will mainly use the eA3A-based CE-CGBE for subsequent optimizations.

### 2.2 Cas-embedding CGBE editors fused with different functional proteins

Previous research identified factors that could enhance CGBE editing efficiency, such as RBMX, Udgx, and POLD2 [9]. To verify the effect of these factors on CE-CGBE editors, we first fused Udgx and RBMX into the CE-CGBE editors. Using the Anc689 deaminase, we constructed four types of Cas-embedding CGBEs (Fig. S2A). Each of the five editors along with their respective sgRNAs were transfected into HEK293T cells, GFP-positive cells were sorted by flow cytometry, DNA was extracted, and deep sequencing was performed to analyze editing efficiency. The results showed that for Anc689-based Cas-embedding CGBEs, editing efficiency was generally lower compared to the control group (U-Anc-U-nCas9-R) at most sites (Fig. S2B). We also replaced Anc689 with the rAPOBEC1 deaminase to prepare another four types of CGBE editors. Similarly, the Cas-embedding CGBEs exhibited reduced efficiency compared to the control. Next, we fused POLD2 and Udgx into the CE-CGBE editors using the rAPOBEC1 deaminase, constructing four types of CGBEs (Fig. S3A). These five types of editors also did not yield the desired results (Fig. S3B).

**Fig. 3:**
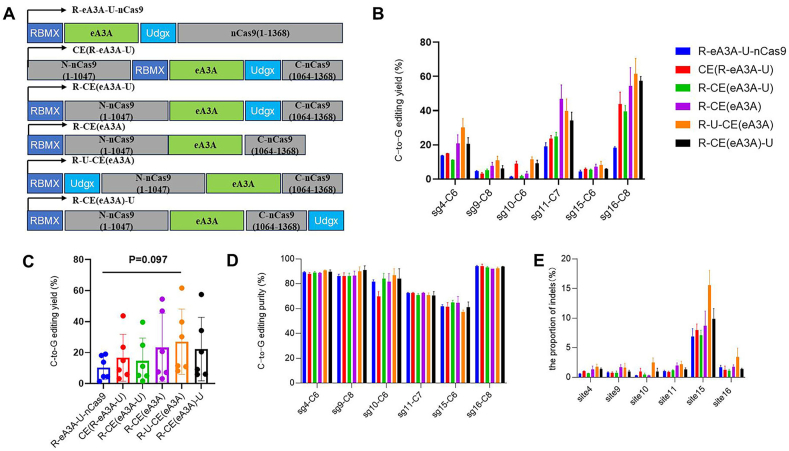
**Editing Characteristics of Various Types of Cas-Embedding CGBE Constructed Based on eA3A.** (A) RBMX and Udgx were fused with eA3A-based CGBE editors in different forms. The Cas-embedding editors involve eA3A replacing residues 1048–1063 in nCas9. (B) Editing efficiency of Cas-embedding CGBE editors fused with RBMX or Udgx at multiple target sites. Only the highest editing efficiency points within the target sites were counted. Each sample was replicated three times. (C) Summary of editing efficiencies for different types of CGBE editors, where each point represents the editing of a target site. The values in the graphs are mean ± s.e.m. The statistical test used was the Mann-Whitney *U* test. (D) Editing purity of different types of CGBE editors. Each sample was replicated three times. (E) Indel generation by different types of CGBE editors. Each sample was replicated three times.

Using RBMX and Udgx, we constructed another six types of CGBEs based on the eA3A deaminase, naming them R-eA3A-U-nCas9, CE(R-eA3A-U), R-CE(eA3A-U), R-CE(eA3A), R-U-CE(eA3A), and R-CE(eA3A)-U (Fig. 3A). These six editors along with their respective sgRNAs were transfected into HEK293T cells, GFP-positive cells were sorted by flow cytometry, and DNA was extracted. Deep sequencing analysis of editing efficiency showed that among six target sites, R-U-CE(eA3A) had significantly improved editing efficiency compared to the control group (R-eA3A-U-nCas9) at all six sites (Fig. 3B). We then calculated the average editing efficiency of the six CGBE editors (Fig. 3C), showing that R-U-CE(eA3A) had editing efficiency close to a significant level compared to the control (p = 0.097) (Fig. 3C). We also analyzed the purity of the six CGBE editors, finding that the editing purity remained consistently high for all six CGBEs (Fig. 3D). Analysis of indels revealed that Cas-embedding forms of CGBEs showed an increase, particularly for R-U-CE(eA3A), which reached around 15 % at site 15, but remained low at the other five sites (Fig. 3E).

### 2.3 Off-target analysis of CE-CGBE

**Fig. 4:**
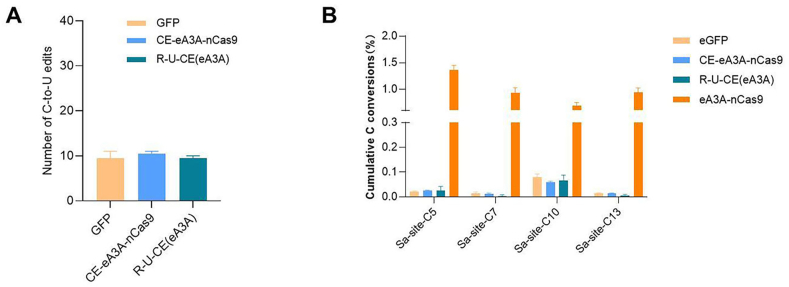
**Analysis of Off-Target Effects for Cas-Embedding CGBE Constructed Based on eA3A.** (A) RNA off-target analysis, with GFP as the control. Each sample was replicated three times. (B) Analysis of random DNA off-target effects of the CGBE editor using the R-loop method. Each sample was replicated three times.

The Cas-embedding base editing construction strategy involves better integration of the deaminase with Cas9 to reduce the likelihood of the deaminase randomly contacting irrelevant ssDNA. Next, we will analyze the off-target effects of the optimized CE-CGBE editors. We selected two Cas-embedding forms of CGBE, CE-eA3A-nCas9 and R-U-CE(eA3A). The respective CGBE editors were co-transfected with sgRNA targeting site 16 into HEK293T cells, and strongly GFP-positive cells were sorted, followed by extraction of both DNA and RNA. First, we predicted sgRNA dependent off-target sites using Cas-OFFinder software and selected four of them for deep sequencing [21]. The results showed that, compared to the control, CE-eA3A-nCas9 and R-U-CE(eA3A), did not exhibit significant changes in sgRNA-dependent DNA off-targeting (Fig. S4). Next, using RNA sequencing, we analyzed RNA off-targeting. The results showed that, compared to the negative control group, CE-eA3A-nCas9 and R-U-CE(eA3A) produced off-target effects at the RNA level consistent with background levels, The results showed that, compared to the Ea3a-nCas9 group, CE-eA3A-nCas9 and R-U-CE(eA3A) significantly reduced RNA off-target effects, bringing them down to background levels (Fig. 4A).

For cytidine deaminase-mediated editors, R-loop method is an unbiased analysis approach used to examine sgRNA-independent off-targeting caused by deaminases [22]. The R-loop method utilizes another types of Cas systems, such as saCas9, which bind to specific DNA sequences to create ssDNA [22]. If off-targeting occurs with the CGBE base editing based on spCas9, it theoretically produces off-targets on the ssDNA. R-loop method has been widely applied in sgRNA independent off-targets analysis and serves as a perfect alternative to whole-genome sequencing methods [[23], [24], [25]]. The saCas9 system was co-transfected into cells along with different versions of CGBE that we constructed. GFP and mCherry double-positive cells were then isolated. Following amplification of the target sequences, deep sequencing was conducted to identify sgRNA-independent off-target effects. The results indicated that, compared to the negative control group, CE-eA3A-nCas9 and R-U-CE(eA3A) did not exhibit significant off-targeting among the four cytidine bases of the sgRNAs used (Fig. 4B). The above data suggest that CGBE base editors constructed using the Cas-embedding strategy maintain high fidelity and represent a highly pure, safe, and efficient CGBE system. Considering the editing efficiency, purity and indels, the R-U-CE(eA3A) editor is the optimal CGBE editor we have selected so far, which we have named HF-CGBE (High fidelity CGBE).

### 2.4 Utilizing HF-CGBE to mimic hereditary pathogenic mutation

**Fig. 5:**
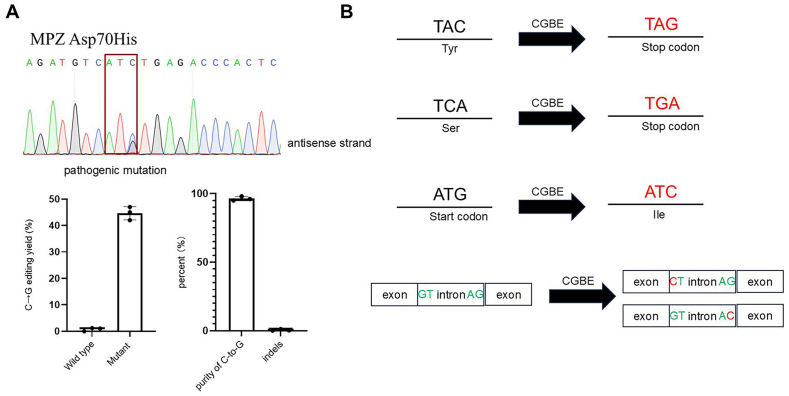
**Applications of CGBE Base Editors.** (A) Simulation of genetic pathogenic mutations using CGBE base editors. The 70th amino acid of the MPZ gene is mutated from C to G, changing the amino acid Asp to His. Using the R-U-CE(eA3A) editor, we achieved a simulated mutation at this site with an editing efficiency greater than 40 %, and purity exceeding 95 %. The indel ratio was below 1 %. Each sample was replicated three times. (B) Strategies for gene knockout using CGBE. These include inducing stop codons, disrupting the start codon, and disrupting variable splicing sites.

According to currently reported data on hereditary pathogenic mutations, approximately 11 % of such mutations require correction using CGBE base editors [26]. To validate the role of HF-CGBE in hereditary pathogenic mutations, we selected a reported pathogenic mutation site, the c.178G > C mutation in the MPZ gene, which causes the 60th amino acid to change from Asp to His. This kind of mutation is associated with axonal Charcot-Marie-Tooth (CMT) disease [27]. We produced this mutation using HF-CGBE in HEK293T cells. The results showed that the efficiency of using HF-CGBE to edit this mutation reached around 45 %, and we also analyzed the purity of the editing, finding that 95 % of the edits were C-to-G (Fig. 5A). Additionally, we detected that the proportion of indels at this site could be controlled to less than 1 %. Precision is crucial when simulating or correcting pathogenic mutations, and this case demonstrates the value of HF-CGBE in simulating pathogenic mutations.

## 3 Discussion

Base editing, due to its ability to efficiently edit target genes without causing significant double-strand breaks in DNA, is a safer editing tool and has already been widely used. While base editing may sometimes lead to a low frequency of DSBs, these events can be significantly reduced through optimization of the editing conditions. Additionally, because base editing does not cause significant chromosomal translocations when editing multiple sites and has minimal impact on cell proliferation [28], it holds great potential for development in the fields of cellular and gene therapy [29]. BEAM-101, a base-editing therapy for sickle cell disease, has been recognized as one of the "eleven clinical trials that will shape medicine in 2025" [30].

CGBE is a base editor that converts C to G, similar to cytosine base editors, which also utilize cytidine deaminases. However, since base editors incorporate deaminases, they can produce sgRNA independent off-target effects, which is an aspect that needs special attention when developing products using base editing [12,13]. Previous studies have indicated that placing the deaminase as a functional domain in the flexible region of the Cas9 protein can significantly reduce sgRNA independent off-target [15]. The Cas-embedding strategy has been extensively utilized with a variety of Cas enzymes, such as saCas9 [17,31], Nme2Cas9 [23,24], and CasRx [32]. In these diverse applications, the Cas-embedding approach has shown its effectiveness in reducing off-target editing. It is reasonable to speculate that the Cas-embedding strategy could also be applied to a broader range of Cas enzymes, including Cas12 and Cas13 proteins. Beyond minimizing off-target effects, this strategy also contributes to enhanced stability and increased production yields of the editing proteins [25].

This study utilized the Cas-embedding strategy to construct various types of CGBE editors. CE-CGBE showed no significant differences compared to controls in terms of editing efficiency and editing window, although it could improve editing efficiency at certain sites. We also observed that the editing efficiency of CGBE varied significantly across different sites, indicating that it is challenging to develop a universal CGBE to meet most needs. Further optimization revealed that the CE-CGBE editor incorporating RBMX and Udgx (HF-CGBE), showed editing efficiency approaching significance compared to controls (p = 0.097) and achieved an editing purity of around 80 %. More importantly, HF-CGBE results in significantly fewer sgRNA-independent off-target effects compared to the control group, aligning closely with the negative control. Precision is a critical consideration in gene and cell therapy, and with further optimization, HF-CGBE has the potential to achieve editing efficiencies similar to those of ABE or CBE [18].

The applications of CGBE are extensive. In addition to repairing hereditary pathogenic mutations, CGBEs have many other applications. One of the most important is their ability to introduce point mutations to achieve gene knockout. Previous studies have utilized CBE to induce stop codons, while ABE and CBE have been used to disrupt variable splicing sites and the start codon [33]. CGBEs can also achieve gene knockout by inducing stop codons. By acting on TAC (Tyr) or TCA (Ser) sites, CGBEs can induce the formation of stop codons, TAG or TGA, respectively (Fig. 5B). Additionally, CGBEs can act on the start codon ATG, inducing it to become ATC, which disrupts normal protein translation. Finally, CGBEs can also act on variable splicing sites, converting GT/AG into CT/AC, disrupting normal mRNA splicing to achieve the purpose of gene knockout.

In summary, using the Cas-embedding strategy, we have provided more efficient and precise HF-CGBE editing tools, expanding the gene editing toolkit and offering new avenues for basic scientific research and the development of gene and cell therapy drugs.

## 4 Materials and methods

### 4.1 Plasmid construction

The expression sequences for the POLD2, RBMX, and Udgx genes were synthesized by GenScript (Nanjing) following codon optimization. The vector assembly was carried out using the ClonExpress II One Step Cloning Kit (Vazyme, C112-01). The DNA oligos required for constructing the sgRNA expression vectors were synthesized, annealed, and ligated into the BsaI-digested pGL3-U6-sgRNA-EGFP vector. The detailed sgRNA sequences used for detecting on-target editing activities are provided in Table S1.

### 4.2 Cell culture and transfection

HEK293T cells were cultured in DMEM supplemented with 10 % fetal bovine serum (v/v) (Gibco) and 1 % Penicillin-Streptomycin. Cells were incubated at 37 °C with 5 % CO₂. For plasmid transfections, HEK293T cells were seeded on 24-well plates one day prior to transfection and transfected at approximately 70 %–80 % confluence. Two days post-transfection, GFP-positive cells were harvested via fluorescence-activated cell sorting (FACS). To assess the editing activities of CGBE, HEK293T cells were transfected with 600 ng of editors and 300 ng of corresponding sgRNAs. To evaluate RNA off-target effects, HEK293T cells were seeded in a 6-cm dish and transfected with 4 μg of editors and 2 μg of sgRNAs, followed by isolation of GFP-positive cells. To detect sgRNA-dependent off-target DNA editing, 600 ng of editors were co-transfected with 300 ng of corresponding sgRNAs, and GFP-positive cells were collected three days later. SgRNA-dependent off-target sites were predicted using Cas-OFFinder software (http://www.rgenome.net/cas-offinder/), and four of the potential off-target sites were analyzed (Table S2).

### 4.3 Deep sequencing

Sorted cells were lysed in a buffer containing 50 mM KCl, 1.5 mM MgCl_2_, 10 mM Tris (pH 8.0), 0.5 % Nonidet P-40, 0.5 % Tween 20, and 100 mg/mL protease K. Fragments containing the target sites were amplified using the cell lysates as templates. PCR products were analyzed via Sanger sequencing and deep sequencing on the Illumina NovaSeq 6000 platform (2 × 150 PE). Sanger sequencing results were further processed using EditR (https://moriaritylab.shinyapps.io/editr_v10/). Deep sequencing data were processed as previously described. Briefly, AdapterRemoval version 2.2.2 was used to remove adapter sequences from paired-end reads; paired-end reads aligning to 11 base pairs or more were combined into a single consensus read. The BWA-MEM algorithm (BWA v0.7.16) was then used to map the processed data to the target sequences. Indels were considered to occur in reads containing at least one inserted or deleted nucleotide within the protospacer.

### 4.4 RNA sequencing

For the assessment of RNA off-target editing, HEK293T cells isolated by FACS were immediately treated with TRIzol reagent, and total RNA was extracted according to the manufacturer's instructions. RNA sequencing was performed on the Illumina NovaSeq 6000 platform (2 × 150 PE) at a depth of ≥10 million reads per sample. RNA sequencing data were processed as previously described. Briefly, the STAR software (Version 2.5.1) was used to map sequence reads to the human reference genome (hg38); GENCODE version V30 was utilized for annotation. After removing duplicates, variants were called using GATK HaplotypeCaller (version 4.1.2) and filtered with QD (quality by depth) <2. Edits were required to have at least 10× depth and at least 99 % of reads supporting the reference allele in wild-type samples. Finally, only C-to-G edits in the transcribed strand were used for further analysis.

### 4.5 R-loop

Orthogonal R-loop assays, used to analyze sgRNA-independent off-target DNA editing, were conducted as previously described. Briefly, 300 ng of tested editors, 200 ng of sgRNAs, 300 ng of nSaCas9 (5′ GTGGCACTGCGGCTGGAGGT3′), and 200 ng of SaCas9 sgRNAs were co-transfected into HEK293T cells. Three days post-transfection, GFP and mCherry double-positive cells were isolated. The sgRNA expresses GFP, and the saRNA expresses mCherry.

### 4.6 Statistics

The results from two or three independent experiments were presented as the mean ± s.e.m. Statistical analysis and graphing were performed using GraphPad Prism 8.0. Student's two-sided *t*-test or Mann-Whitney *U* test was used for comparisons, and p < 0.05 was considered statistically significant.

## CRediT authorship contribution statement

**Tian Lin:** Writing – original draft, Data curation. **Xin Wang:** Data curation. **Yu Zhang:** Methodology, Funding acquisition. **Guanglei Li:** Writing – review & editing. **Xingxu Huang:** Project administration. **Ming Shi:** Writing – review & editing, Investigation.

## Declaration of competing interest

The authors declare that they have no known competing financial interests or personal relationships that could have appeared to influence the work reported in this paper.
