# Supplementary material for: Developing safe and efficient CGBE editor based on Cas-embedding strategy
Source: Synth Syst Biotechnol. 2025 Feb 6;10(2):504–10. doi: 10.1016/j.synbio.2025.02.001 (PMC11872432; doi:10.1016/j.synbio.2025.02.001)
Supplement: Multimedia component 1 [file mmc1.docx]

**Developing Safe and Efficient CGBE editor Based on Cas-embedding strategy**

**SUPPLEMENTARY INFORMATION**

Figure S1 C-to-G Editing Purity and Indel Proportions of CGBE Editors Mediated by Different Deaminases.

Figure S2 Editing Efficiency of Cas-Embedding CGBE Fused with Udgx and RBMX.

Figure S3 Editing Efficiency of Cas-Embedding CGBE Fused with POLD2 and Udgx.

Figure s4 sgRNA-Dependent DNA Off-Target by Cas-Embedding CGBE Editors Based on the eA3A Deaminase.

Table S1 The target sequencing of the sgRNA

Table S2 The predicted off-target sites for sgRNA16


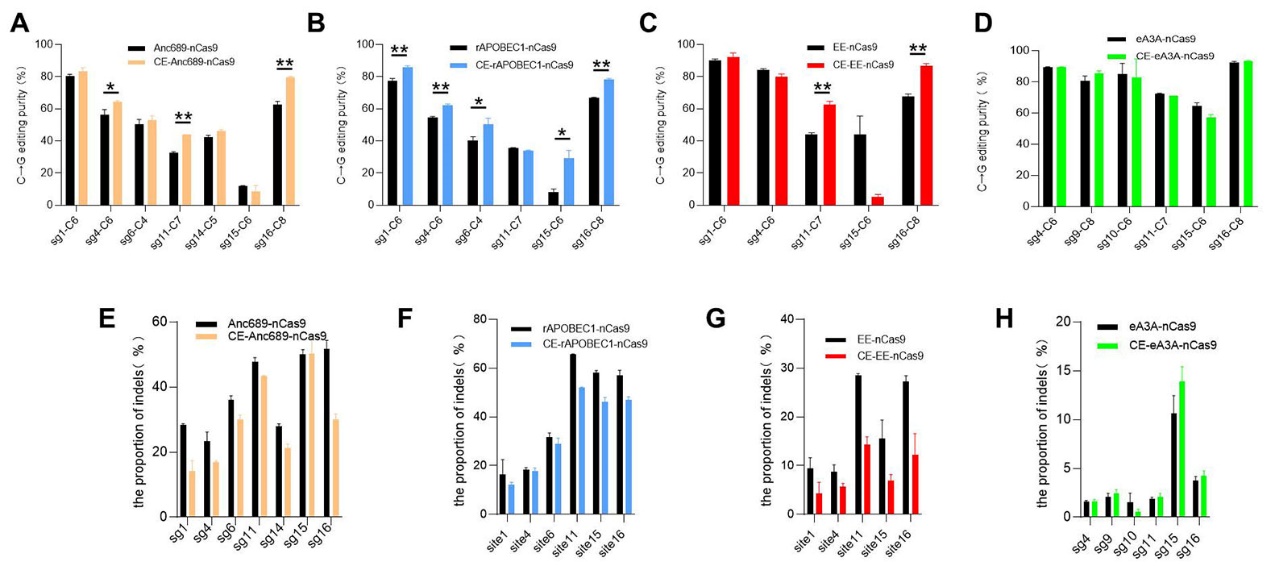


**Figure S1 C-to-G Editing Purity and Indel Proportions of CGBE Editors Mediated by Different Deaminases.** (A-D) C-to-G editing purity at different editing sites for CGBE editors mediated by four types of deaminases. (A) is for Anc689 deaminase, (B) for rAPOBEC1 deaminase, (C) for EE deaminase, and (D) for eA3A deaminase. * indicates significant differences, ** indicates highly significant differences. Each sample was replicated three times. The statistical method used was Student’s two-sided t-test.(E-H) Proportion of indels generated at different targets by CGBE editors mediated by four types of deaminases. (E) is for Anc689 deaminase, (F) for rAPOBEC1 deaminase, (G) for EE deaminase, and (H) for eA3A deaminase. * indicates significant differences, ** indicates highly significant differences. Each sample was replicated three times. The statistical method used was Student’s two-sided t-test.


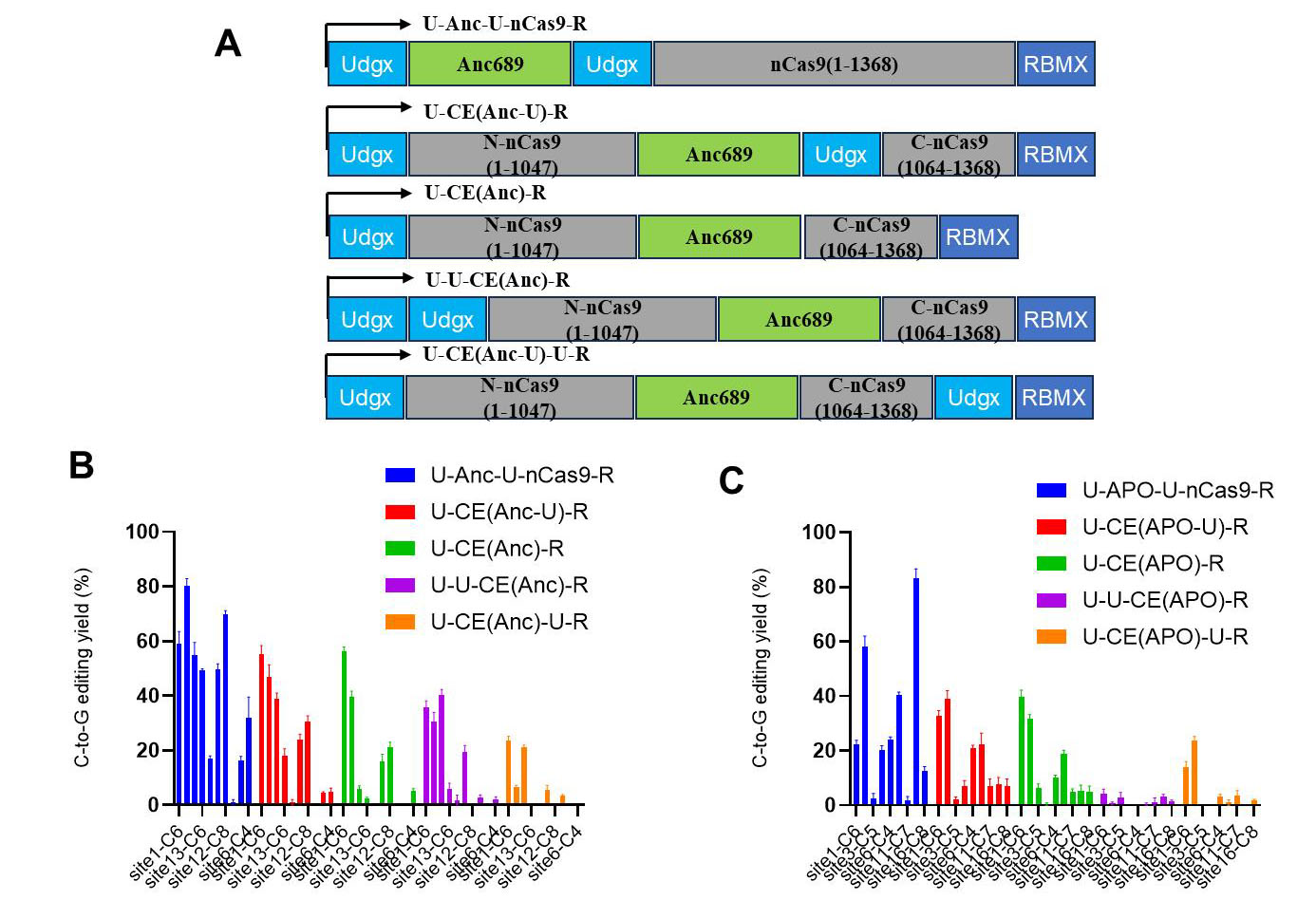


**Figure S2 Editing Efficiency of Cas-Embedding CGBE Fused with Udgx and RBMX.** (A) Udgx and RBMX fused with Cas-embedding CGBE in different forms.(B) Editing efficiency of various types of CGBE based on the Anc689 deaminase. Each sample was replicated three times.(C) Editing efficiency of various types of CGBE based on the rAPOBEC1 deaminase. Each sample was replicated three times.


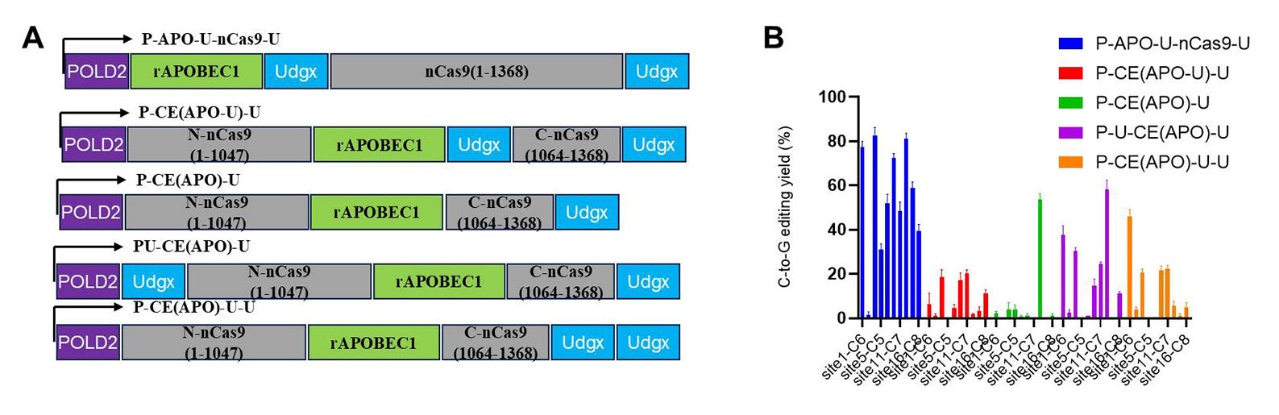
**Figure s3 Editing Efficiency of Cas-Embedding CGBE Fused with POLD2 and Udgx.** (A) POLD2 and Udgx fused with Cas-embedding CGBE in different forms.(B) Editing efficiency of various types of CGBE based on the rAPOBEC1 Deaminase. Each sample was replicated three times.


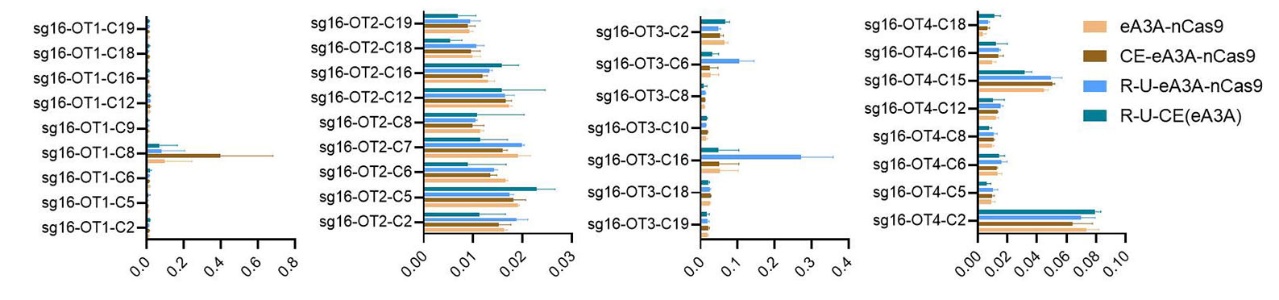


Figure S4 sgRNA-Dependent DNA Off-Target by Cas-Embedding CGBE Editors Based on the eA3A Deaminase. Each sample was replicated three times. The values on the horizontal axis indicate the proportion of C mutations to D (where D represents G, A, or T).

| site | sgRNA（5’→3’） | site | sgRNA（5’→3’） |
| --- | --- | --- | --- |
| Site1 | GAACACAAAGCATAGACTGC | Site2 | GGCCCAGACTGAGCACGTGA |
| Site3 | GGCACTGCGGCTGGAGGTGG | Site4 | GTCATCTTAGTCATTACCTG |
| Site5 | GAGTCCGAGCAGAAGAAGAA | Site6 | CCTCCAGCCGCAGTGCCACC |
| Site7 | GCACTTGTTTGCAGCTATTC | Site8 | GAGCTAACTGTGACAGCATG |
| Site9 | TGCTTCTCCAGCCCTGGCCT | Site10 | GCCATCTTGAAGGGAGGGGA |
| Site11 | CGTGCTCAGTCTGGGCCCCA | Site12 | GCACATACTAGCCCCTGTCT |
| Site13 | CCCTTCAAGATGGCTGACAA | Site14 | AGCACGTGATGGCAGAGGAA |
| Site15 | GGAATCCCTTCTGCAGCACC | Site16 | GCAACCTCAAACAGACACCA |

Table S1 The target sequencing of the sgRNA

Table S2 The predicted off-target sites for sg16

| site | Off-target sequence（5’→3’） | site | Off-target sequence（5’→3’） |
| --- | --- | --- | --- |
| sg16-OT1 | aCAtCCTCcAACAGACACCAGGG | sg16-OT2 | aCAACCcCAgACAGACACCAAGG |
| sg16-OT3 | GCAcCtTCAtACAGACACCAAGG | sg16-OT4 | GCAAaCTCAcAaAGACACCAGGG |
